# Building resilience to climate risks through social protection: from individualised models to systemic transformation

**DOI:** 10.1111/disa.12339

**Published:** 2019-04-04

**Authors:** Martina Ulrichs, Rachel Slater, Cecilia Costella

**Affiliations:** ^1^ Program Officer, Pathy Family Foundation Canada; ^2^ Professor of International Development, Centre for International Development and Training University of Wolverhampton United Kingdom; ^3^ Senior Technical Advisor, Red Cross Red Crescent Climate Centre

**Keywords:** cash transfers, climate, Ethiopia, Kenya, resilience, social protection, Uganda

## Abstract

This article analyses the role of social protection programmes in contributing to people's resilience to climate risks. Drawing from desk‐based and empirical studies in Ethiopia, Kenya and Uganda, it finds that social transfers make a strong contribution to the capacity of individuals and households to absorb the negative impacts of climate‐related shocks and stresses. They do so through the provision of reliable, national social safety net systems—even when these are not specifically designed to address climate risks. Social protection can also increase the anticipatory capacity of national disaster response systems through scalability mechanisms, or pre‐emptively through linkages to early action and early warning mechanisms. Critical knowledge gaps remain in terms of programmes’ contributions to the adaptive capacity required for long‐term resilience. The findings offer insights beyond social protection on the importance of robust, national administrative systems as a key foundation to support people's resilience to climate risks.

## Introduction

Climate‐related risks, including disasters, are understood to be one of the biggest threats to social and economic progress, with particularly detrimental effects on poverty and inequality (Hallegatte et al., 2016). To effectively build resilience to disasters, the international community has called for a more integrated approach that seeks to link disaster response with longer‐term reduction of vulnerability to climate change. The aim is to prevent climate hazards from becoming disasters in contexts of chronic poverty and food insecurity. Social protection is seen as a critical ingredient in resilience‐building agendas, because of its demonstrated impact on poverty reduction through the provision of consumption support and the protection of vulnerable people from the impoverishing impacts of different risks. The Sendai Framework for Disaster Risk Reduction 2015–2030 specifically highlights the need to promote and support the development of ‘*social safety nets as disaster risk reduction measures linked to and integrated with livelihood enhancement programmes in order to ensure resilience to shocks at the household and community levels*'. The humanitarian community is also increasingly exploring using social protection systems as mechanisms to deliver humanitarian assistance faster and more effectively (HLPHCT, 2015).

Consequently, demand for ‘shock‐responsive', ‘climate‐smart’ and ‘adaptive’ social protection systems is on the rise (Davies et al., 2009; Kuriakose et al., 2012; OPM, 2015). Development partners and governments in many countries in Asia, Latin American and sub‐Saharan Africa are seeking to adjust social protection systems to be more climate‐sensitive, or embedding capacity to address short‐term shocks, as well as long‐term stresses, in newly designed programmes.

This research aimed to critically analyse which elements of existing large‐scale national social protection programmes in Ethiopia, Kenya and Uganda contribute to the capacity of individuals and national systems to absorb, anticipate and adapt to climate‐related shocks and stresses. It assessed safety net programmes that specifically aim to build resilience to climate‐induced humanitarian crises, as well as those designed to support categorically vulnerable groups such as old people and children.

This article is structured as follows. The next section outlines the resilience framework adopted for the study and highlights the potential contributions social protection can make to building resilience capacities. We then summarise the key findings from the case study research on social protection programmes in Ethiopia, Kenya and Uganda. The article concludes with a discussion of the policy implications for social protection and resilience‐building interventions that aim to reduce disaster risks.

## Conceptual framework and methodology

### Framing resilience and social protection using the 3As model

Analysis of the relationship between social protection and resilience to climate risks has emerged steadily over the past decade. Noteworthy examples include the work led by the Institute for Development Studies on ‘adaptive social protection’ (Davies et al., 2009; Béné et al., 2013), by the World Bank on climate‐responsive social protection (Kuriakose et al., 2012) and by the Africa Climate Change Resilience Alliance on social protection, climate change and disaster risk reduction (Jones et al., 2010), as well as a burgeoning body of work on how far social protection programmes can be flexible and scalable to respond to climate shocks (see Bastagli, 2014; Barca and O'Brien, 2017; and O'Brien et al., 2018 for substantial overviews and case studies).

While social protection has the potential to contribute to resilience, particularly given the imminent threat climate change poses to achieving the Sustainable Development Goals of no poverty, at the same time a number of challenges emerge. The analysis presented here originates in recognition of these challenges, especially those concerning the framing of the resilience and social protection agenda, which we seek to critically examine. Our inquiry emerges from both research and preparatory work by the authors themselves (Slater and Bhuvanendra, 2013; Slater et al., 2015; Ulrichs and Slater, 2016; Holmes and Costella, 2017) and the evidence in the bodies of work noted above and others.

Three assumptions underlie the framing of social protection and climate resilience narratives, which this research aimed to unpack. First, extreme weather events rather than longer‐term trends in climate variability and seasonality are foregrounded in conceptual and policy frameworks and studies on social protection and resilience. This in turn leads to a focus on shock‐responsive social protection, and questions about whether emergency and humanitarian responses can piggyback on existing social protection systems. Sometimes, this can be at the expense of assessments of the long‐term outcomes of social protection programmes for mitigating the (human‐made) underlying causes of vulnerability, such as soil degradation and falling agricultural productivity and incomes, or for long‐term human capital development. As Johnson et al. (2013) note, the ability of social protection programmes to build livelihoods and resilience in the long term in a context of a changing climate remains poorly understood and risks being sidelined by the dominance of a shock‐responsive agenda.

Second, there is an assumption that social protection programmes in general, and unconditional cash transfer programmes in particular (that are not designed specifically to help people manage climate risks), cannot contribute much to resilience. While there is evidence that cash‐based support through safety nets has been insufficient to buffer beneficiaries from extreme climate shocks (Devereux and Guenther, 2009), the application of this evidence has, as yet, been rather unrefined. Basic cash transfers have been viewed as limited in their potential to provide shock response or build long‐term resilience without wider programmatic linkages (Innocenti, 2016; FAO, 2017; Roelen et al., 2017). There has been no nuanced assessment of the duration, reliability and adequacy of transfers vis‐à‐vis extent, severity and type of shock, nor any sustained effort to understand how strengthening the delivery of (often fledgling) social protection programmes might enhance their effectiveness during periods of shocks.

As a result, the emphasis for social protection is largely on programmes that specifically aim to address climate risks by either (1) scaling up in response to a short‐term shock episode or (2) incorporating more complex elements beyond the transfer, such as asset‐building through public works or savings and loans to enable households to build assets and transform livelihoods (described as ‘social protection plus’ or productive safety net programmes). In the programming space between these two ends of a spectrum, ranging from emergency response to long‐term livelihood transformation, far less attention is paid to how social protection can contribute to resilience. This represents a major knowledge gap that undermines effective programming.

The third assumption relates to the conceptualisation of resilience, which often emphasises building resilience at the individual level at the expense of system and structures. Social protection tends to be viewed as supporting resilience because of the outcomes and impacts it is expected to generate for individuals and their households as a result of programme support—specifically the capacity to independently withstand shocks and stresses—rather than outputs (e.g. income support for households) and participation in the programme being the source of resilience. To better understand the relationship between social protection and resilience, it is also necessary to uncover whether programmes build resilience or whether people are more resilient because they can fall back on a programme. As Slater and Bhuvanendra (2013) suggest, people are resilient because they are beneficiaries in social protection programmes and not just because they have built their autonomous capacity to withstand shocks without assistance. Following from this assumption, assessments of social protection and resilience have tended to prioritise the question of whether social protection increases people's capacity to withstand shocks in the absence of support, over the question of whether it is necessary to strengthen social, governance and administrative systems in order to address the underlying causes of vulnerability—including those not related to climate change (O'Brien et al., 2004; Cannon and Müller‐Mahn, 2010; Harrison and Chiroro, 2017).

The desire to address these concerns in our exploration of the relationship between social protection and resilience in Ethiopia, Kenya and Uganda led us to draw on the ‘3As’ model of resilience to frame our analysis. While resilience is often understood as an outcome that can be measured and monitored, it has been increasingly acknowledged that a more useful way of conceptualising resilience is as an ability (Béné et al., 2012). In general, resilience in a policy context is defined mostly as the ability to anticipate, avoid, plan for, cope with, recover from and adapt to (climate‐related) shocks and stresses. Bahadur et al. (2015) have broken down this concept into three key capacities—absorptive, anticipatory, and adaptive (the 3As). This approach can facilitate the analysis and design of programmes that aim to build resilience by contributing to these capacities.

**Absorptive capacity** allows people or systems to absorb and cope with climate‐related shocks and stresses while and after they occur. It enables people to reduce the immediate negative impact on livelihoods and basic needs. **Anticipatory capacity** enables people and systems to be better prepared for the eventuality of a specific shock through proactive action by avoiding or reducing exposure or by minimising vulnerability to the shock. **Adaptive capacity** is understood to be the ability to adapt to multiple and long‐term climate risks, as well as the ability to learn and adjust after a disaster to reduce vulnerability to similar shocks in the future.

The 3As framework can be put to work to assess social protection. It is possible to distil from existing analysis some of the potential resilience capacities resulting from social protection that can be analysed through the framework. Béné et al. (2012) analysed the overlaps between the key functions of social protection (prevent, protect, promote and transform (Devereux and Sabates‐Wheeler, 2004)) and the three resilience capacities (then defined slightly differently, as absorptive, adaptive and transformative). Their aim was to explore whether social protection programmes contributed to strengthening the resilience of beneficiaries, and, if so, through which capacity. They argued that, in theory, while cash transfers could potentially be used to adapt or transform livelihoods, in fact their safety net—that is, absorptive—function was the bedrock of any resilience‐building intervention.

Social protection programmes that contribute to building absorptive capacity at the individual level include safety nets, collective loans or savings schemes and weather‐indexed insurance: all assist households in meeting their consumption needs in the immediate aftermath of a hazard. Kenya's Hunger Safety Net Programme (HSNP) and Mexico's conditional cash transfer, for example, allow people to absorb shocks without engaging in negative coping strategies (De Janvry et al., 2004; Merttens et al., 2013).

Anticipatory capacity can be demonstrated in the ability of communities to manage disaster risks by planning in advance, for example through disaster response plans, training exercises and natural resource management. Ethiopia's Productive Safety Net Programme (PSNP) and Kenya's HSNP are frequently cited as successful examples of flexible and scalable cash transfer programmes that can provide an emergency response in times of need, albeit with relatively little robust empirical analysis to support this claim (the next section explores these examples as case studies). Similarly, ‘social protection plus’ programmes, whereby cash transfers are complemented with asset development, skills training and access to microcredit and microfinance, have the potential to support household adaptive capacity—for example the transition into livelihoods that are less exposed to climate‐related shocks.

We use the 3As model to specifically investigate the contributions to the three capacities of two different types of programmes: (1) safety nets that aim explicitly to reduce vulnerability to climate‐related risks (often including public works to generate assets and other programme components); and (2) categorically targeted safety nets (such as old age pensions and child grants). The latter were included to challenge the assumption that social protection programmes need to be specifically designed to address climate risks to contribute to resilience. Looking at the two types of programmes also allowed us to distinguish between the basic functions of cash and asset transfer programmes, versus their explicit objectives. The 3As model thus enables an assessment of the contributions of social protection that does not privilege or assume one type of programme is more impactful than another but rather allows for a robust and comprehensive analysis across a range of programme types to identify which social protection design elements make a difference to resilience capacities.

Using the 3As model also allows us to differentiate between these capacities at the individual/household and systems level. The initial attraction of the term ‘resilience’ for development programming stemmed from its systemic, multi‐scalar approach, which captured the range of shocks individuals and communities are exposed to, as well as the processes and dynamics that affect the socio‐ecological system across scales, from local to global (Béne et al., 2012). Some of this systems approach has been muted, as research and policy attention has switched to a focus on impacts on individuals and households. Nevertheless, using the 3As allows us to interrogate the nature of the systems—particularly how they might support anticipatory capacity through contingency financing, pre‐registration of households at risk of exposure to shocks, etc., but also how long‐term social protection systems might tackle the vulnerabilities that reinforce the negative impacts of shocks. The 3As also allow us to be clear when we need to think about resilience capacities, for example in different stages in the policy and project cycle—from problem analysis, to the allocation of budgets and departmental mandates, to the design and implementation of programmes—and to identify where social protection's contributions to resilience capacities can be strengthened.

## Methodology

A desk‐based review of existing evidence in the three case study countries was combined with primary qualitative research in Kenya and Uganda at the national policy level, as well as at the district level and with participants and non‐participants in social protection programmes in villages in Turkana (Kenya) and Apac (Uganda).

The desk‐based review of social protection policies and programme documents allowed us to identify the underlying theories of change of the individual programmes and how/whether the programmes formed part of the national strategies to respond to climate shocks and disasters. Key informant interviews (KIIs) at the national level in Nairobi and Kampala and at sub‐national level with representatives of government ministries, development partners and civil society organisations provided data on perspectives of the role social protection plays in the national resilience agenda and how it contributes (in theory and practice) to building people's resilience capacities. In selected counties and districts in Turkana (Kenya) and Apac (Uganda), focus group discussions (FGDs) were conducted. These complemented existing evaluation data with qualitative data to better understand how beneficiaries and non‐beneficiaries in the selected field sites had dealt with climate‐related shocks in the past, based on a participatory risk ranking exercise. To account for gender differences and dynamics, we conducted separate FGDs with men and women, as well as mixed‐group FGDs. In‐depth interviews were also conducted with female and male beneficiaries to allow for a better understanding of the household‐level impact of shocks and programme participation.

The qualitative research aimed to answer the three overarching questions:

1. Does participation in a programme increase people's capacity to *absorb* the negative impacts of climate shocks without suffering setbacks in their well‐being?
2. Does participation in the programme allow beneficiaries to take any measures to prepare for and *anticipate* the eventuality of a shock?
3. Does current or past participation in a programme increase people's capacity to *adapt* their livelihoods to reduce their vulnerability to future climate risks?

The selection of social protection programmes in each country (Table 2) allowed for a number of comparisons: between basic cash transfer programmes and those with more complex delivery systems (such as those including public works); between programmes with climate change‐related or resilience objectives and those with other objectives (addressing poverty and food insecurity or supporting specific social protection); between government and donor‐driven programmes; and between more established and fledgling programmes.

**Table 1:** Overview of methods

	Kenya	Uganda	Total
KIIs national level (government, non‐governmental/research organisation, development partner)	11	12	23
KIIs county/district level	7	6	13
In‐depth interviews beneficiaries (half with men/half with women)	10	11	21
FGDs (half with men/half with women)	6	8	14

**Source:** authors.

**Table 2:** Overview of programmes

Programme	Description
**Kenya**	
Cash Transfer for Orphans and Vulnerable Children (CT‐OVC)	The CT‐OVC was launched in 2004 as one of the first government‐run and ‐financed cash transfers in Kenya, with support from development partners including the UN Children's Fund (UNICEF), the Swedish Agency for International Development Cooperation, the UK Department for International Development (DFID) and the World Bank. It was a direct response to the growing AIDS pandemic that was eroding informal family and communal coping mechanisms.
	The CT‐OVC is an unconditional cash transfer to poor households caring for OVC with the aim of improving welfare and reducing poverty. It operates in all 47 counties and benefits 255,643 households, of which the government finances 215,470 (MLEAA, 2016).
Hunger Safety Net Programme (HSNP)	The HSNP was launched in 2007 and is an unconditional cash transfer that aims to reduce poverty in counties in northern Kenya. The National Drought Management Authority (NDMA) implements the programme under the Ministry of Devolution and Planning. In its second phase (2013–2017), the HSNP was to a large extent still funded by development partners, with the aim to progressively increase the share of government funding to cover over 50% of the transfer costs.
	The HSNP is currently reaching out to 84,340 households in four counties (Turkana, Marsabit, Mandera and Wajir) with the objective of expanding coverage to an additional 100,000 households (MLEAA, 2016).
**Uganda**	
Social Assistance Grants for Empowerment (SAGE)	SAGE forms part of the government's Expanding Social Protection programme, and has piloted two cash transfer programmes: the Vulnerable Family Support Grant and the Senior Citizen Grant (SCG).
	The pilots were implemented through the Ministry of Gender, Labour and Social Development with funds and technical support from DFID, Irish Aid and UNICEF.
	Both SAGE pilots together aimed to reach 560,000 people in 124,547 households over a period of four years (2011–2015), covering approximately 15% of households in 14 districts. From 2015, only the SCG was continued. The SCG is currently in the process of gradual expansion to new districts.
Northern Uganda Social Action Fund (NUSAF)	NUSAF is the largest public works programme in Uganda, with approximately 77,000 beneficiaries in 2013 (McCord et al., 2013). It consists of a combination of public works, household asset transfer programmes and community infrastructure. In its third phase, NUSAF 3 (2015–20) aims to increase the provision of seasonal productive safety nets and link it to disaster risk financing to allow scalability following a shock.
	The programme operates through two different implementation modalities, in Karamoja (World Food Programme) and in the remaining northern counties (Oxford Policy Management) with support from development partners.
**Ethiopia**	
Productive Safety Net Programme (PSNP)	The PSNP is part of the government of Ethiopia's Food Security Programme and provides seasonal public works programmes for poor, chronically food‐insecure able‐bodied households. The PSNP has been in place since 2005. As part of the integrated Risk Financing Mechanism (RFM), the PSNP delivers additional assistance to food‐insecure people affected by unpredicted shocks. In its fourth phase, the PSNP currently supports close to 8 million people.

**Source:** authors.

The amount of time dedicated to empirical data collection was limited to 10 days per country (four days in the national capital, one day in the district/county capital and four days in villages in Turkana/Apac). Given the very short amount of time per site, findings from this research cannot be considered representative, and they are not indicative of programme impact. The fieldwork rather asked context‐specific questions to better understand the relevance of climate shocks for people in different localities as well as the perspectives of beneficiaries and non‐beneficiaries as to whether social protection programmes had allowed them to respond to such shocks.

## Findings

### Resilience contributions at the individual level

Findings from the three country case studies highlight that national social protection programmes currently make a strong contribution to people's capacities to absorb the negative impacts of climate‐related shocks and stresses on their livelihoods. They do so through the provision of well‐implemented, regular cash or in‐kind transfers—regardless of whether these aim specifically to address climate or lifecycle‐based risks.

The PSNP and HSNP, for instance, are programmes that aim to specifically reduce vulnerability to drought of chronically and transitorily food‐insecure households. Both programmes have had a positive impact on food insecurity indicators (such as increasing the number and size of meals) of routine beneficiaries (Maxwell et al., 2013; Merttens et al., 2013). Few evaluations have found that the protective function of these safety nets is maintained during times of extreme shocks (Farhat et al., 2017). Routine recipients are able not only to smooth consumption during the food gap period but also to maintain their asset levels and bounce back faster than non‐beneficiaries after periods of extreme drought (Maxwell et al., 2013; Knippenberg, 2016). A study conducted by Knippenberg (2016) on the PSNP suggests it ‘*reduces vulnerability [to a drought] by 60% and doubles the level of resilience, significantly improving the post‐treatment recovery trajectory. … When a household experiencing drought receives the mean level of PSNP payments (498 birr, approximately $23), their welfare drops less following a shock and recovers more rapidly*'. An evaluation of the emergency scale‐up of the HSNP found that people who received temporary emergency support managed to meet their basic needs but negative coping strategies such as distress sale of assets were not prevented (Farhat et al., 2017).

While these impacts are to be expected from safety nets implemented specifically to address drought‐induced food insecurity, there is currently limited evidence suggesting categorically targeted programmes fulfil a similar function. The qualitative primary research conducted in Kenya and Uganda aimed to fill this gap through consultations with recipients of child grant programmes and old age pensions, as well as through consultations with non‐recipients.

In Turkana, the research found that recipients of the CT‐OVC used transfers in a very similar way to HSNP recipients. That is, they used income from transfers largely to meet their basic consumption needs. Considering that only 14% of the county population engages in agro‐pastoralism, the food security of the majority of the population depends on their ability to purchase imported food in the market. In both programmes, the main use for the cash is food, and the impacts in terms of real household consumption are similar, with an increase by KSh 274 (USD 3.40) per adult per month on average in the CT‐OVC equivalent against and increase in the HSNP of KSh 247 (USD 2.40) (Ward et al., 2010; Merttens et al., 2013). While evaluations of the CT‐OVC did not assess how the use of cash transfers differed depending on whether there was a drought or not, during our qualitative research respondents did indicate that the cash was used nearly exclusively for food during extreme droughts whereas it was used more frequently for non‐food expenses the rest of the year.

In Apac in Uganda, a district categorised as highly vulnerable to flooding and prolonged dry spells (UNDP, 2016), respondents indicated that use of the SAGE transfers differed depending on the season. In food‐secure months, it is used for a range of household expenses, such as food, school fees or health care. As in Merttens et al. (2015), households reported that the transfer was a key safety net allowing them to meet their basic food needs in the lean season or following drought‐induced food insecurity.

The regular provision of monthly or bimonthly cash transfers thus provides households with the flexibility to use the cash according to changing priorities and needs. In food‐secure months, they can invest the cash in assets, savings or education; when faced with shocks, such as sickness or drought‐induced food insecurity, it protects the household from potential income losses. Across sites, recipients mentioned saving either in cash or through buying livestock to be better prepared for unforeseen expenditures, such as health issues or food shortage from own production owing to drought. This resonates with evaluation findings on Uganda's SAGE, where a statistically significant increase was observed in the proportion of beneficiary households having savings, which beneficiaries explicitly perceived as a strategy to mitigate risks and generate cash in times of need (Merttens et al., 2015). In Turkana cash transfer recipients use it to similarly prepare for the eventuality of a shock, by sharing the transfer and thus investing in their informal networks (which explains the high levels of dilution of the transfer: 25% of beneficiaries reported sharing at least some of their transfers with others (Merttens et al., 2013)). This is an action taken in advance of a crisis, in anticipation of having to resort to these resources during times of hardship, and is a pattern also observed among pastoral groups in Ethiopia receiving PSNP support (Sabates‐Wheeler et al., 2013). This highlights that regular cash transfers give recipients the flexibility to plan ahead for shocks, through the purchase of assets or through savings, which increases the anticipatory capacity of households, whereas the ability to draw on these assets or the transfer following shocks increases their absorptive capacity.

In contrast, Uganda's temporary public works programme NUSAF 2 (2010–14) does not provide cash over several months or years but rather one‐off cash for work to food‐insecure households over a limited period of 22 days. If timed correctly, such temporary safety nets allow households to absorb shocks by protecting consumption and alleviating acute food insecurity. The short‐term nature of the NUSAF 2 assistance, as well as its unreliable payments and the timing of the public works activities, means it is akin more to ad hoc emergency relief than to regular safety nets (McCord et al., 2013). The contributions short‐term public works programmes can have on resilience capacities are thus different from those of regular cash transfers through safety nets. The short‐term employment can (at best) provide temporary absorptive capacity but has limited impact on anticipatory or adaptive capacity. The resilience outcomes are expected to result from the community assets built, which include natural resource management or conservation activities and aim to reduce vulnerability to climate hazards. Despite widespread support for public works programmes as resilience interventions, evidence to substantiate the impact of community assets on livelihood resilience is very limited and shaky (McCord, 2013).

In sum, findings from the three country case studies highlight that national, well‐implemented social protection programmes that provide regular cash transfers have the largest potential to contribute to the 3As—regardless of whether they aim to reduce categorical vulnerability linked to a lifecycle risk (old age/childhood) or to reduce vulnerability to climate risks. This, in turn, challenges the assumption that social protection needs to be explicitly designed to assist people in managing climate risks to build resilience. The contributions to absorptive capacity are the clearest, as safety nets provide a buffer to absorb shocks when they occur, including climate‐related ones. They also contribute to some extent to individual anticipatory capacity (such as through the accumulation of savings), but impact on adaptive capacity is extremely limited to non‐existent. Based on respondents’ views, the only potential long‐term resilience impact emerging from the regular cash transfers is their contribution to school fees, which can enable the next generation to access paid employment. In terms of mid‐term adaptive capacity, the size of the cash transfer is too small to make any larger investments to transform livelihoods. Further access to key assets and services needed to shift into more adaptive livelihoods is missing, such as access to climate information, irrigation systems or skills and training in climate‐smart practices. This highlights the need for complementary interventions in other sectors that can provide social protection beneficiaries with the necessary assistance to build more resilient livelihoods in the long term.

### Resilience contributions at the systems level

Using the 3As allows us to see the different types of impact social protection can have on resilience capacities at the systems level too (Table 3). While the impacts of categorically targeted and climate‐sensitive programmes on routine beneficiaries are similar, the shock‐responsive element of the latter makes significant additional contributions to the anticipatory capacity of systems in strengthening national disaster preparedness and response. Flexible and scalable programmes can expand assistance quickly to people affected by a serious shock—even before the impacts materialise. To do so, adequate trigger, funding, targeting and delivery mechanisms need to be in place. This anticipatory capacity of the system can then enable timely and preventative disaster response, which in turn increases individuals’ absorptive capacity once a shock hits. By helping avoid negative coping strategies, this early action may play a critical role in protecting development gains and help build resilience (RCCC, 2017).

**Table 3:** Resilience outcomes at the individual and systems level

Household/individual level	Institutional/systems level
**Absorptive**	
Cash transfers allow people to meet basic consumption needs even during times of shocks.	Putting in place effective delivery mechanisms that can deliver assistance even during times of crisis.
Assets and savings accumulated through cash transfers provide buffers.	
**Anticipatory**	
Cash transfers provide people with the ability to save in anticipation of a shock.	Putting in place delivery mechanisms, forecast‐based financing or contingency funds, and operational procedures (including trigger, target group) to deliver assistance through social protection programmes in anticipation of or shortly after a disaster.
**Adaptive**	
Enabling households to improve their livelihoods through asset‐building and income generation activities that are less vulnerable to climate risks.	Providing linkages between social protection and other livelihood programmes.

**Source:** authors.

The HSNP and PSNP link existing cash transfer delivery and targeting mechanisms with national disaster response. The HSNP was designed as a permanent safety net for chronically vulnerable households, but from the outset sought to establish procedures and contingency funds that would allow it to become an effective response mechanism in case of emergency and to build anticipatory capacity at the systems level. This included targeting and delivery mechanisms that covered chronically food‐insecure ‘routine’ recipients but also worked to allow scale‐up through the preidentification and registration of households vulnerable to food insecurity in the case of an extreme or severe drought. Having operational guidelines in place for the scale‐up of HSNP assistance in case of a drought, triggered using an objective and quantifiable Vegetation Condition Index, enables the system to deliver assistance within 10 days of declaring an emergency. This has increased the capacity to respond significantly in HSNP areas: humanitarian aid used to take three to nine months to reach beneficiaries (NDMA, 2016).

The PSNP also, in its second phase (2010–2015), put in place a Risk Financing Mechanism (RFM), which enabled horizontal and vertical expansion during times of crisis through financial pre‐positioning of contingency funds (Hobson and Campbell, 2012). A World Bank review in 2013 estimated that assistance through the PSNP in response to the food crisis in the Horn of Africa in 2011 was cost‐efficient, at $53 per beneficiary, compared with $169 through the UN‐coordinated humanitarian response. The key elements considered decisive for disaster response through social protection are (1) effective early warning systems, (2) contingency plans that define triggers for emergency response and roles and responsibilities, (3) earmarked contingency funding and (4) institutional arrangements and capacity to deliver (Hobson and Campbell, 2012).

While the examples from the PSNP and HSNP make a convincing argument for using social protection to deliver a more timely and cost‐efficient disaster response, they are only one part of the picture in managing climate risks systematically. To reduce the burden of shocks on vulnerable populations and increase the cost effectiveness of support, acting earlier, even before the shock has happened, may be crucial (Costella et al., 2017). Many climate‐related hazards such as storms, floods and droughts can be predicted, often including estimates of their location, intensity, probability and duration. When combined with complementary information such as on exposure and vulnerability, it is possible to identify populations at risk of being affected before a disaster strikes (RCCC, 2017). There are documented advantages to acting early to respond to climate shocks and disasters, including avoiding disaster losses and increasing cost effectiveness (Ebi et al., 2004; Braman et al., 2013; Coughlan de Perez et al., 2015; Pappenberger et al., 2015).

Social protection can integrate early action and preparedness to support more effective resilience‐building at scale. However, few systematic experiences have focused on designing and implementing programmes to help households and governments *anticipate* such shocks (Ulrichs and Slater, 2016), for instance by linking to or setting up early warning early action systems (triggers and contingency planning) to respond as soon as a shock happens or even before it does (Costella et al., 2017).

Based on the experience of forecast‐based financing (FbF), a recent paper (Costella et al., 2017) explores whether social protection can be linked to these approaches from the humanitarian sector. FbF is a mechanism that enables early warning systems to take early action measures based on pre‐agreed forecast triggers and protected funding. Several early actions can be taken based on forecast information, selected on the basis of their effectiveness, such as prepositioning of relief items, distribution of goods, etc. Successful activation of forecast‐based cash transfers or other ‘shock‐responsive’ actions depends on the capacity of the system to pre‐identify beneficiaries, as well as to execute distribution in the short window of time between a forecast and occurrence of the hazard.

This highlights the need for administrative systems to demonstrate resilience capacities at different moments in time from design to implementation, as well as to build linkages between information and delivery systems across sectors. For example, putting in place funds, early warning and targeting systems for emergencies in advance to allow social protection to scale up is inherently anticipatory since it enables better preparedness by avoiding or reducing exposure and vulnerability. This anticipatory capacity can then enable the programme to meet its absorptive (or protective and preventive) function more adequately in times of crisis. This is not to say that absorptive capacity necessarily has to be preceded by anticipatory capacity. Regular cash transfers, for example, are delivered without necessarily anticipating a specific shock over a longer period of time. Despite not being explicitly anticipatory, they still provide people with the capacity to absorb shocks when they happen.

Going back to the assumptions laid out at the start, this finding underlines not only how programmes contribute to the resilience capacities of individuals but also that they can make critical contributions to strengthening administrative systems at the national level to improve and institutionalise the provision of assistance for vulnerable groups, as well as to strengthen national disaster response systems. This is an important contribution to acknowledge and build on. Having resilient systems in place that can address the chronic and transitory needs of people exposed to climate risks needs to be a critical aspect of any comprehensive strategy to reduce vulnerability to climate risks.

### Programme form versus programme function

Looking across the various programmes and the contributions to the 3As they make with regard to resilience capacity at the individual and system level, it is striking how much similarity there is in the form of programmes, in terms of their design and implementation mechanisms, and how much diversity in their objectives—that is, the functions they seek to provide.

NUSAF and the PSNP, for example, both combine interventions of consumption support through short‐term or seasonal cash for work with asset‐building (at the community and household level). The HSNP, CT‐OVC and SAGE, on the other hand, provide nearly the same amount of cash on a regular basis to poor households, yet based on different eligibility criteria and pursuing different objectives. Analysis across these programmes allows us to reflect on the relative importance of their form (this includes design and implementation) versus their function, or stated objective, as well as the importance of coherence between the two and the capacity to implement.

First, when it comes to objectives, two similar programmes in terms of form—the HSNP and CT‐OVC—differ in their objectives and policy narratives. The HSNP forms part of the key resilience policy framework on Ending Drought Emergencies and is managed by the NDMA. It was designed specifically to address recurring food crises in drought‐prone areas to reduce the risk of recurring humanitarian crises. The CT‐OVC, on the other hand, is implemented by the Ministry of Labour, Social Security and Services and was put in place in the mid‐2000s as a response to the HIV/AIDS crisis (Pearson and Alivar, 2009). According to key informants, the different focus of cash transfer programmes is often linked to political economy factors, such as higher political acceptance of programmes targeting specific groups over others, as well as dominant discourses and funding streams in the donor community.

Despite these differences in institutional homes and overarching objectives, they hardly differ in what they do or in their impact. Both deliver a similar amount of cash to vulnerable households. Impact evaluations suggest both support recipients to meet their consumption needs, facilitate school attendance of children, pay for health care and purchase small livestock (Ward et al., 2010; Merttens et al., 2013). Similarly, SAGE in Uganda aims to economically empower categorically vulnerable groups through a regular income transfer and—just as in the CT‐OVC in Kenya—is not framed around a resilience narrative yet leads to absorptive outcomes and potentially supports adaptation.

This highlights that programmes that have a similar form contribute to a similar extent to resilience capacities, regardless of whether they specifically aim to do so. This is not to say that resilience objectives are irrelevant or that they do not influence programme delivery. However, the HSNP appears to outperform the CT‐OVC because of its more efficient and timely payment delivery infrastructure, through bank cards and mobile agents, rather than because of the transfer itself (or the specific objective to reduce drought vulnerability).

Compared with basic cash transfers, NUSAF covers a more complex range of interventions, and it has shifted its objectives from supporting peace and reconciliation in NUSAF 1, to improving infrastructure and access to basic services in NUSAF 2, to building resilience in NUSAF 3 (McCord et al., 2013; see also Republic of Uganda, 2015). Its core activities, however, have remained more or less the same, with a combination of short periods of public works, asset transfers and improving community infrastructure and assets. Furthermore, given lack of evidence on the impacts of the assets created or rehabilitated through NUSAF 2, as well as the short‐term nature of income support provided through the public works, SAGE in Uganda arguably has a stronger impact on resilience capacities than NUSAF 2—and yet does not state, either explicitly or implicitly, the intention to do so.

The wider development literature provides some insights into the challenges that emerge when programmes’ form and function appear to be relatively disconnected. Pritchett et al. (2010), exploring how institutions often find themselves caught in capability traps that undermine the transition to improved developmental governance, identify a phenomenon they call ‘isomorphic mimicry'. In this, development actors copy the form of other organisations or institutions but not the functions that those original forms are meant to deliver. It appears, when we consider programmes in Kenya and Uganda, that something similar may be present in social protection, where either highly similar forms of programme are expected to play very different roles or functions, or the functions of a single programme change remarkably over time, with unremarkable shifts in the form of the programme.

Social protection's capacity to contribute to the 3As can be compromised if coherence between the objective, programme design and implementation is missing. This is particularly critical in more complex programmes, such as cash for work, which aim to combine short‐term consumption support with long‐term resilience‐building through community assets. Here, the evidence suggests that a missing link between programme objective, design and implementation means that the desired outcomes are unlikely to be achieved (such as NUSAF, McCord et al., 2013). While the objective of categorically targeted cash transfers is not primarily climate resilience, the design process should still include assessments of what and how climate risks might need to be integrated into their design and implementation. This will help ensure that the impacts of the programme are not undermined and that the programme does not lead to unintended consequences that might increase vulnerability in the long term (for instance, increase reliance on unsustainable livelihoods or maladaptive practices such as exploitation of natural resources in Ethiopia (Weldegebriel and Prowse, 2013)). Last but not least, good implementation is critical for programme impact. In some cases, good implementation may by default contribute to the 3As, such as in the case of well‐implemented categorically targeted cash transfers.

## Conclusion and implications for policy and programming on resilience‐building

This research aimed to critically analyse some of the emerging framings of resilience as well as social protection's role in strengthening people's capacity to anticipate, absorb and adapt to climate‐related risks. Unpacking some of the assumptions made about social protection and its role leads to a range of policy implications for social protection, as well as for resilience policies and programmes more widely.

### Implications for social protection as a resilience‐building tool

First, a focus on increasing response to disasters caused by extreme weather events has dominated discussions around social protection's role in providing more effective disaster response. Findings from the research highlight the important contribution well‐established social protection programmes can make in increasing the capacity of national disaster response systems. There is significant scope to refine and improve social protection programmes that have the capacity to scale up, for instance by linking contingency funds to forecast‐based trigger mechanisms to ensure assistance is delivered in anticipation of a shock to prevent disasters from even happening.

As relevant as it is, a primary focus on extreme events and disasters risks losing sight of the need to address the underlying vulnerability that increases exposure to climate hazards. The provision of reliable safety nets is necessary in contexts of chronic poverty and vulnerability, as demonstrated by the evidence on how unconditional cash transfers increase people's capacity to absorb and anticipate climate shocks.

Second, there is limited evidence that there is the capacity to design and implement national multi‐component, highly complex programmes in those countries that exhibit the twin features of high exposure to climate‐related shocks and high levels of poverty and vulnerability. However, this does not mean we have to choose between them. The Ethiopia PSNP example shows there is potential to incorporate more complex social protection programming, including a broader set of objectives around climate change, but that this takes time, and requires high or increasing levels of capacity and substantial technical assistance and cooperation between governments and their development partners.

The key policy implication that emerges from this finding is that policy‐makers and implementers in government, international development actors and national civil society organisations are likely to make more progress towards building resilience through social protection by focusing first on getting the basics of national social protection programmes right. This includes, for example, concentrating on delivering transfers regularly and reliably and on scaling up sustained coverage of poor and vulnerable people. A premature focus on expanding the functions and technical scope of programmes, and the addition of auxiliary features, can be damaging—particularly if the commitment to long‐term financial and technical support is not guaranteed (either by the government or by development partners). It can lead to what Pritchett et al. (2010) have called ‘premature loadbearing’ or ‘asking too much too soon of too little too often’ of new and fledgling programmes that have limited coverage and relatively low levels of capacity. In turn, this can subsequently undermine the achievement of core programme objectives and, most dangerous of all, result in waning support expenditure on social protection.

At the same time, focusing on improving the structural form of programmes rather than simply changing their headline objectives should be a priority for governments. Challenges emerge when institutional form and function are poorly aligned (where development actors mimic the form of another organisation or institution but do not require the same functions). This ‘isomorphic mimicry’ (as coined by Pritchett et al., 2010) is evident in social protection, where either highly similar forms of social protection are meant to meet very different objectives or the functions of a single programme change substantially over time and yet there are few accompanying changes in the programme design.

### The need for a systems approach to resilience policies and programming

The research highlights that resilience requires the provision of permanent systems that support vulnerable individuals and communities in the case of extreme events. The current approach of many resilience‐building initiatives is heavily focused on building people's autonomous resilience capacities, to be sustained in the absence of external support. Livelihood programmes focusing on building the 3As capacities include a complex range of interventions in a limited timeframe, after which people are expected to be able to fend for themselves, or receive short‐term support in the case of an emergency. The focus on building adaptive capacity through a combination of interventions is attractive because it allows for an exit strategy from projects and programmes (that is, that a combination of support will allow households to move into independent and sustainable livelihoods in which they can buffer themselves against shocks and stresses). And, for non‐governmental organisations (NGOs) specifically (which rarely deliver cash transfers at national scale), the focus on adaptation allows them to deliver complex, multi‐pronged social protection/asset transfer/livelihoods projects through which they can achieve reasonably high coverage in a small geographical space.

The resilience impacts achieved by the different programmes in the study are the result of years, if not decades, of trial, error and creeping progress. Efforts by national governments, development partners and NGOs have gradually strengthened national delivery systems and expanded linkages to other sectors, such as disaster risk reduction, through early warning and early action mechanisms. However, where programmes aim to include a complex set of interventions within a tight timeframe and project‐specific design, there is little evidence of success in building the long‐term resilience of participants. To strengthen resilience at the individual and household level, it is thus critical to understand how national systems that span different sectors at the national and sub‐national level can be transformed to provide reliable and sustainable support structures that reduce vulnerability to livelihood risks in the long term.

Doing so might imply different roles for governments, development partners and NGOs, recognising there is greatest value in focusing mainly and initially on reducing vulnerability by allowing people to anticipate and absorb shocks, at scale, through government programmes, using the broader activities of NGOs to plug gaps and being ready to provide lessons to government as it increases capacity and improves delivery. When governments are able to transition to more complex programming, there are practical lessons from NGO experience on what works for them to draw on.
